# Plasticization Effects of PEG of Low Molar Fraction and Molar Mass on the Molecular Dynamics and Crystallization of PLA-*b*-PEG-*b*-PLA Triblock Copolymers Envisaged for Medical Applications

**DOI:** 10.1021/acs.jpcb.5c00171

**Published:** 2025-03-20

**Authors:** Nikolaos D. Bikiaris, Panagiotis A. Klonos, Evi Christodoulou, Panagiotis Barmpalexis, Apostolos Kyritsis

**Affiliations:** † Department of Pharmaceutical Technology, School of Pharmacy, Aristotle University of Thessaloniki, GR-541 24 Thessaloniki, Greece; ‡ Dielectrics Group, Department of Physics, National Technical University of Athens, Zografou Campus, GR-15780 Athens, Greece; § Department of Chemistry, Laboratory of Polymer Chemistry and Technology, Aristotle University of Thessaloniki, GR-541 24 Thessaloniki, Greece

## Abstract

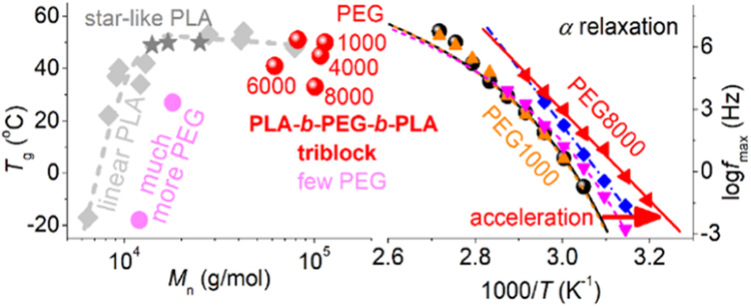

We prepared and studied a series of triblock copolymers based on poly(ethylene glycol) (PEG) and poly(lactic acid) (PLA). PLA blocks were *in situ* by ring-opening polymerization (ROP) of lactide (LA) onto the two sites of PEG. While in our recent work on similar copolymers with varying LA/PEG molar ratios and fixed PEG blocks [BikiarisN. D.Mater. Today Commun.2024, 38, 107799], herein, we kept this ratio quite low, at 640/1, and employed different molecular weights, *M*_n_, of the initial PEG at 1, 4, 6, and 8 kg/mol. The triblocks demonstrated high homogeneity, as manifested by the single thermal transition (glass transition, crystallization) with corresponding alternations in a systematic way with the *M*_n_ of PEG. With the increase of the latter *M*_n_, accelerated segmental mobility and lowering of *T*_g_ by up to 15 K were recorded, accompanied by suppression in the chain fragility (cooperativity). Compared with linear PLAs of various *M*_n_s [KlonosP. A.Polymer2024, 305, 127177] and other PLA-based copolymers prepared by similar ROPs, with the overall *M*_n_ of our copolymers, PEG here sees to play the role of plasticizer on PLA, leading to increased free volume. Due to these effects, in general, the low crystalline fraction of PLA (∼3%) was significantly enhanced in the copolymers (20–26%), and the formed spherulites were mainly enlarged. Contrary to these, nucleation was barely affected; thus, the copolymers exhibited altered semicrystalline morphologies as compared to that in neat PLA. Both aspects of molecular dynamics, free volume and crystallization, were connected to the processability as well as the performance of these systems, considering the envisaged biomedical applications.

## 1 Introduction

Polymers and polymer-based materials have served humanity for many decades,^1,2^ being implemented in almost all aspects of everyday life, medicine, industry, and academia. These materials have conquered the market, replacing traditional materials such as metals and wood in many applications, as they combine easier/milder manufacturing processes and reproducibility with good performance and low economic cost. The most widely used polymers, such as polystyrene, polyethylene, and poly(methyl methacrylate), have been basically synthesized from fossil-based resources employing relatively nonecofriendly routes. The massive use of synthetic polymers (plastics) has resulted in a severe environmental threat. An extensive accumulation of plastics in nature has been proved, mainly in the sea and the soil, which form the so-called “plastic waste”.^3,4^ As expected, the world’s needs constantly change, seeking materials for new applications, sustainability, and fulfilling recent environmental consciousness.^5,6^ For the past two decades, we have faced a turn toward the green and circular economy,^7,8^ within which a significant number of polymers synthesized from renewable resources via more eco-friendly methods have been developed. This class is characterized now as “green and sustainable polymers”.^9−12^ Among them are the quite known polyesters poly(lactic acid) (PLA)^13−15^ and poly(*ε*-caprolactone) (PCL).^16^ More recently, novel renewable polyesters have been presented, being synthesized from acids coming from natural biological processes (e.g., metabolism), such as succinic, furan-dicarboxylic, adipic, vanillic, and azelaic acids.^4,17−20^

PLA is mainly prepared from starch, sugar cane, and beet,^21,22^ and can be synthesized on a large scale via relatively mild thermochemical methods, e.g., ring-opening polymerization (ROP).^23^ This polyester is probably the most known and applied renewable polymer to this day. This is because PLA exhibits a sum of quite wanted characteristics, namely, good thermochemical stability,^24^ and mechanical performance.^25^ Its performance is strongly related to its semicrystalline character,^24^ while due to the moderately low glass transition (*T*_g_) and melting (*T*_m_) points,^26−29^ PLA offers possibilities for manipulations of the crystallinity degree and semicrystalline morphology.^28,30−33^ PLA has been, additionally, shown to be compatible with other polymers, thus favoring the preparation of polymeric blends, copolymers, and polymer networks.^34−39^ By manipulating the structure of PLA, e.g., the molecular weight and d-/l-lactide ratio, along with the addition of ‘softer polymers’ to PLA, *T*_g_ can be tuned from ∼30 to ∼70 °C, whereas *T*_m_ from ∼140 to 190 °C.^28,31,33,40−42^ Such a combination of characteristics makes PLA an almost ideal material; therefore, it is already used in numerous applications, both traditional and modern (packaging, biomedicine, 3D printing, etc.).^43−47^

Unfortunately, bulky PLA lacks biodegradation under soil/sea conditions, which has been rationalized via the concept of slow physical hydrolytic cleavage of ester bonds in these environments.^48−50^ The factors precluding fast hydrolytic/enzymatic depolymerization in PLA are mainly its rigid structure (glassy with a small free volume fraction), hydrophobicity, and semicrystallinity. The issue has been faced by combining PLA with other renewable polymers (mainly polyesters) in the form of polymeric blends and copolymers.^37,39,51−55^ The addition of a lower *T*_g_ component combined with a proper mixing or copolymerization method (e.g., *in situ* polymerization) has been proven quite efficient in the manipulation of the overall performance. In recent works by our groups, involving such systems based on PLA, we presented many results demonstrating the potential for such manipulations.^53,54,56−58^ Among these studies, in Bikiaris et al.,^58^ we prepared and studied a series of triblock copolymers based on PLA blocks in situ (ROP) developed onto low-molecular-weight poly(ethylene glycol) (PEG), PLA-*b*-PEG-*b*-PLA, with PLA/PEG molar ratios ranging from 20/1 to 640/1. The recordings showed the plasticizing role of PEG on PLA and the free volume increase, as manifested by the *T*_g_ from ∼50 to about −40 °C and the suppression of the chain-chain cooperativity, with increasing in the presence of PEG. The crystal nucleation of PLA was facilitated, although the degree of crystallinity was, in general, facilitated. The PLA/PEG ratio and the overall copolymer length were found to be critical parameters affecting the thermodynamic homogeneity as well as the ability to manipulate the semicrystalline morphology to a large extent. These properties are desired as they are useful considering the permeation of small molecules as well as the facilitation of the compostability of PLA. Moreover, such materials are expected to exhibit the so-called thermoresponsive transitions (micelles formation and deformation) in aqueous environments^59−63^ and are envisaged for use in biomedical applications (drug delivery).

In the next step of the latter study, herein, we synthesized and investigated similar PLA-*b*-PEG-*b*-PLA triblock copolymers, keeping the PLA/PEG ratio fixed at 640/1 and altering the molecular weights of the initial PEG block to 1000, 4000, 6000, and 8000 g/mol. The work focuses on glass transition, crystallization, and molecular dynamics. To this aim, we used differential scanning calorimetry (DSC), X-ray diffraction (XRD), polarized light microscopy (PLM), Fourier transform infrared (FTIR) spectroscopy, and broadband dielectric spectroscopy (BDS). To the best of our knowledge, the thermal and molecular dynamics mapping of these materials is shown here for the first time.

## 2 Materials and Experimental Techniques

### 2.1 Materials: Synthesis of PLA-*b*-PEG-*b*-PLA Triblock Copolymers

As mentioned above, based on our previous study on similar systems,^58^ the goal here is to develop PLA blocks via ROP at both the end sites of PEG, keeping the [l-lactide]/[PEG] molar ratio fixed at 640/1 and varying the molecular weight, *M*_n_, of the initial PEG. Since the previously employed route was successful, also for different PLA-based block copolymers,^37,53^ we performed similar thermochemical procedures, which are briefly reported in the following.

The initial block for the copolymer synthesis is PEG with various *M*_n_ values, namely, 1000, 4000, 6000, and 8000 g/mol. PEG as well as stannous octoate (Sn(Oct_2_)), a necessary catalyst, were kindly supplied by Aldrich Co (London, United Kingdom), while high-purity l-lactide was supplied by PURAC Biochem BV (Gorinchem, The Netherlands, actual brand name PURASORBL). PEG and l-lactide in a molar ratio 640/1 were fed into a glass batch reactor along with 400 ppm of Sn(Oct_2_) (as to l-lactide) catalyst dissolved in toluene at a concentration of 20 mg/mL. The reactor was repeatedly evacuated and filled with nitrogen at least three times to remove air as completely as possible. The copolymerization mixture was subjected to heating at 160 °C for 3 h under a nitrogen flow of 5 mm^3^/min, with the stirring rotation speed fixed at 200 rpm. The process was followed by an additional 1 h of heating at 180 °C. In the last step of the process, the reaction chamber was slowly vacuumed for ∼15 min in order to remove any remaining lactide that had not been reacted. Polymerization was terminated by rapidly cooling the flask to room temperature, and white bulk solid products were obtained in all cases. For comparison, neat PLA was also prepared following the same ROP route in terms of thermochemical and time period conditions. So, we prepared and investigated a total of five (5) systems.

Regarding the final form of the samples, for the characterization of solid-state property, disk-like samples were formed by employing thermal pressing and specially prepared metallic molds. Depending on the experimental technique of interest, the disk diameter varied from 15 to 20 mm and the thickness varied from ∼1.5 to ∼3 mm. In all cases, the disk samples in the melt state were rapidly cooled in cold water in an attempt to suppress or even eliminate crystallization and finally obtain amorphous polymers.

### 2.2 Experimental Methods

#### 2.2.1 Fourier Transform Infrared (ATR-FTIR) Spectroscopy

To assess any effects regarding the interactions and direct effects arising from the copolymer structure, i.e., the *M*_n_ of PEG and *M*_n_ of the copolymers, we performed ATR-FTIR measurements on the amorphous samples. The recordings were achieved using an IRTracer-100 spectrophotometer (Shimadzu, Kyoto, Japan) equipped with a QATR 10 Single-Reflection ATR Accessory with a Diamond Crystal. The spectra were recorded in the spectral region of 450–4000 cm^–1^ at a resolution of 2 cm^–1^ for absorbance. Totally, 32 coadded scans were collected and then normalized and baseline-corrected prior to any analysis.

#### 2.2.2 Differential Scanning Calorimetry (DSC)

The glass transition, crystallization, and melting events were assessed by conventional calorimetry using a TA Q200 DSC apparatus (TA Instruments, USA). The instrument was initially well-cleaned and calibrated with sapphires for the heat capacity and indium for the temperature and enthalpy. Samples of ∼7–9 mg in mass were closed in aluminum TZero TA pans. The measurements were carried out in a high-purity nitrogen atmosphere and in the temperature range from −100 to 190 °C. Besides the first heating scan from room temperature up to 190 °C, which was performed to erase the thermal history and optimize the thermal contact between the sample and the pan, two main scans were conducted. *Scan 1* involved fast cooling at nonconstant rates (i.e., 60–100 K/min, employing the command “jump” of the TA software), while *scan 2* involved a slower cooling at 20 K/min. The subsequent heating for both scans 1 and 2 was carried out at a fixed heating rate of 10 K/min.

#### 2.2.3 X-ray Diffraction (XRD)

The crystalline structure and degree of crystallinity were also studied by XRD. The measurements were carried out on semicrystalline samples that had been previously treated as in scan 2 in DSC (melted and cooled at 20 K/min). The diffraction spectra were recorded at room conditions for the 2θ range from 5 to 50°, at a scanning rate of 1 °/min, and employing a MiniFlex II XRD-diffractometer (Chalgrove, Oxford, UK), with Cu Ka radiation for crystalline phase identification (λ = 0.154 nm).

#### 2.2.4 Polarized Light Microscopy (PLM)

To visualize the semicrystalline morphology, PLM images were captured during melt crystallization upon cooling (again, imitating scan 2 in DSC). The images were captured employing a Nikon Optiphot-2 polarizing light microscope equipped with a Linkam THMS 600 heating stage, a Linkam TP 91 control unit, and a Jenoptic ProgRes C10Plus camera with Jenoptik ProgRes CapturePro software.

#### 2.2.5 Broadband dielectric spectroscopy (BDS)

Aiming at studying the direct effects of the copolymer structure on the molecular dynamics by BDS, we started recording on initially amorphous samples. This was achieved as follows: the polymers were inserted between the finely polished brash disks (electrodes, 14 mm in diameter) along with thin silica spacers (100 μm, Novocontrol, GmbH, Germany) and melt-pressed on a hot plate (i.e., out of the BDS system) in order to melt the polymers at *T* well above the melting temperature. In this process, any thermal history is erased, the sample thickness is fixed to 100 μm, and the electrical contact between the polymer and the electrodes is maximized. This sandwich-like capacitor was removed from the hot plate and placed between cold thick metal cylinders, resulting in rapid cooling (nonlinear but fast cooling and >20 K/min) and precluding the polymer crystallization.^64^ Thus, we obtained the polymer in an amorphous and glassy state at room temperature. Subsequently, this capacitor was inserted into the BDS cell and cryostat. The measurement began by cooling the sample as fast as possible down to −150 °C by cold nitrogen gas flow, employing a Novocontrol Quattro cryosystem operating with liquid nitrogen. The measured cooling rate was nonlinear, beginning with ∼22 K/min at higher temperatures and dropping to ∼5 K/min at lower temperatures. During this process, the amorphous state of the polymer is preserved. The sample temperature was stabilized at −150 °C and the measurements began by isothermally recording the imaginary part of dielectric permittivity, *ε″*, related to the dielectric loss as a function of frequency, *f*, in the range from 10^–1^ to 10^6^ Hz. The following are isothermal recordings at gradually increasing *T* in steps of 5 or 10 K, covering the range between −150 and 120 °C. We note that up to ∼80–85 °C, the amorphous character of the polymer is preserved, and this is confirmed by the measurements, e.g., the high magnitude of the segmental relaxation prior to the evolution of cold crystallization.

## 3 Results and Discussion

### 3.1 Structure of the Copolymers

**Figure 1:**
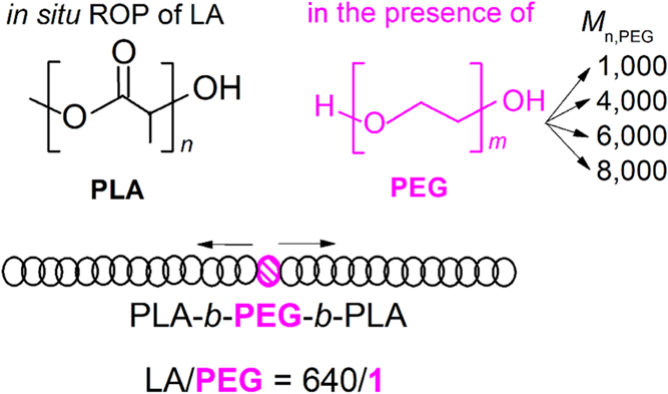
Schematic view of the triblock copolymers based on PLA and PEG under investigation, with the LA/PEG molar ratio fixed and the molecular weight of the initial PEG varying from 1 to 8 kg/mol.

The molecular structures of the newly synthesized materials are shown in Figure 1. There, due to the presence of the −OH groups at both end sites of the PEG block, two PLA blocks can be developed on each PEG block.^58^ In this way, PLA-*b*-PEG-*b*-PLA, or PLA-*tr*-PEG triblocks, can be formed (Figure 1).^58^ Totally, four copolymers at a fixed LA/PEG molar ratio (640/1) and variation of the initial PEG length ranging from 1 to 8 kg/mol were prepared. A comparative study of the copolymers with neat PLA, synthesized under the same reaction conditions, was performed in order to evaluate the direct effect of PEG on the PLA properties and the influence of the different compositions/structures/lengths of the copolymer. The samples are listed in Table 1 along with various values.

**Table 1:** Samples under Investigation, Molar LA/PEG Ratios, and Values of Main Interest^a^

			DSC (initially amorphous, AM)			DSC (semicrystalline, SC)				BDS (amorphous)	
sample	LA/PEG	*M*_n_ (g/mol)	*T*_g,am_ (°C)	Δ*c*_p,am_ (J/g·K)	*T*_cc_ (°C)	*T*_c_ (°C)	Δ*H*_c_ (J/g)	*T*_g,SC_ (°C)	*T*_m_ (°C)	*T*_g,diel_ (°C)	*m*_α_
PLA (neat)	100/0	82k	52	0.48	101	96	2	53	176	51	148
PLA-*b*-PEG(1000)-*b*-PLA	640/1	114k	53	0.53	106	95	2	53	175	50	142
PLA-*b*-PEG(4000)-*b*-PLA	640/1	108k	46	0.58	90	93	8	46	174	45	124
PLA-*b*-PEG(6000)-*b*-PLA	640/1	62k	40	0.59	78	95	24	39	169	41	
PLA-*b*-PEG(8000)-*b*-PLA	640/1	101k	35	0.56	74	91	30	35	166	33	

a *M*_n_, molecular weight of the copolymers and neat PLA; *T*_g,am_ and *T*_g,SC_, glass transition temperatures in the amorphous and semicrystalline states, respectively; *T*_c_ and *T*_cc_, melt- and cold-crystallization temperatures, Δ*H*_c_, melt-crystallization enthalpy change; *T*_m_, melting temperature; *T*_g,diel_, dielectric glass transition temperature; and *m*_α_, fragility index of the segmental *α* relaxation.

**Figure 2:**
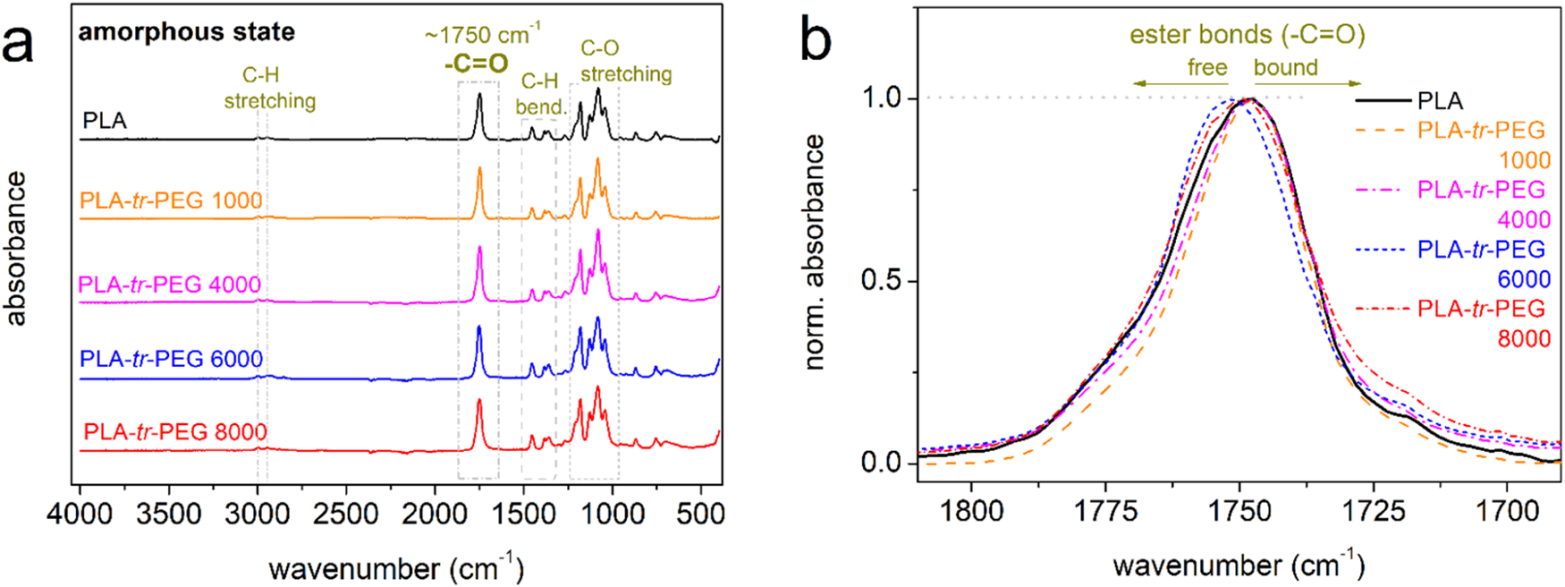
FTIR absorbance spectra for all samples in the amorphous state shown (a) in the overall wavenumber band of the recording and (b) focusing on the FTIR band arising from the ester bond fluctuation.

The formation of PLA by ROP can be initially proven by the FTIR spectra shown in Figure 2. The FTIR profiles for the copolymers are quite similar to that of neat PLA, and this is essential when taking into account the vast 640/1 ratio of LA/PEG. The recorded absorbance originating from the various bond (C–H, C–O, C=O) vibrations is indicated in Figure 2a, in accordance with the literature on PLA.^65,66^ None of the newly formed absorbance bands could be identified for the triblocks from these results.

Among others, FTIR spectroscopy can provide information for newly formed interactions and/or indirect evidence for alternations in the freedom of motion of polymeric groups.^39,67−69^ Regarding PLA and polyesters in general, the ester bond vibration (C=O, at ∼1750 cm^–1^) has been proven to be a useful means for providing such information. This bond is the most polar site of polyesters and, thus, is the most possible candidate to implement within the interchain or other physical interactions of the polymer. For example, when the ester group is ‘engaged’ due to interactions, its absorbance band is enriched at the lower wavenumber side,^67,68^ whereas the existence of unbound C=O results in an absorbance band rich at higher wavenumbers in FTIR spectra. Coming to our case and focusing on the ester group in Figure 2b (shape-normalized FTIR peak), we may observe that compared to neat PLA, in the copolymers, the said FTIR peak seems to be slightly migrated toward higher wavenumbers. Therefore, in the context discussed above, we may expect either fewer occupied ester sites to exist in the copolymers or a significant number of ester groups to exhibit a larger degree of motional freedom.

Based on measurements by gel permeation chromatography–size exclusion chromatography (not shown; methodological details can be found in our recent work^58^), the overall *M*_n_ values of the copolymers were estimated as 114, 108, 62, and 101 kg/mol for PEG1000, 4000, 6000, and 8000, respectively (Table 1). We note that without PEG, the same ROP of lactide resulted in neat PLA with *M*_n_ ∼ 82 kg/mol (Table 1). With the exception of PLA-*tr*-PEG(6000), the increased *M*_n_ of the copolymers compared to that of neat PLA provides a first indication that, indeed, two PLA blocks were formed at both sites of PEG. The fact that the *M*_n_ of PLA was not ‘doubled’ this way can be rationalized considering the fact that the ROP was driven to begin from PEG, whereas, in PLA, it began more randomly onto the initiators. Therefore, the parallel PLA block development toward two directions, i.e., around each PEG block, has resulted in a large *M*_n_; however, it is less than double the *M*_n_ of neat PLA. Regarding the relatively curious case of PEG6000, employing a similar reasoning, we suggest that PEG6000 could play a co-initiator role. This way, in this case, ROP should have begun from many more PEG blocks, resulting in more PLA-*tr*-PEG entities of shorter lengths (lower *M*_n_). Please note that the exceptional behavior of this sample was checked by repeating the synthesis and the measurement of *M*_n_. Moreover, we will show in the following that the lower *M*_n_ is compatible with the corresponding segmental mobility.

Coming back to the FTIR findings on the ester groups, in terms of *M*_n_, the effect of increased freedom of motion in the copolymers is quite compatible with PLA-*tr*-PEG(6000), which exhibits the lowest *M*_n_, whereas it seems less compatible with the rest of the copolymers. Therefore, we may expect additional altered parameters in the structure to be implemented here. Hopefully, the results on molecular mobility will shed more light on this.

### 3.2 Glass Transition in the Amorphous State

Conventional calorimetry (DSC) was employed to assess any effects, primarily on the segmental mobility (glass transition) and, second on the crystallizability (nucleation and formation of crystals).

**Figure 3:**
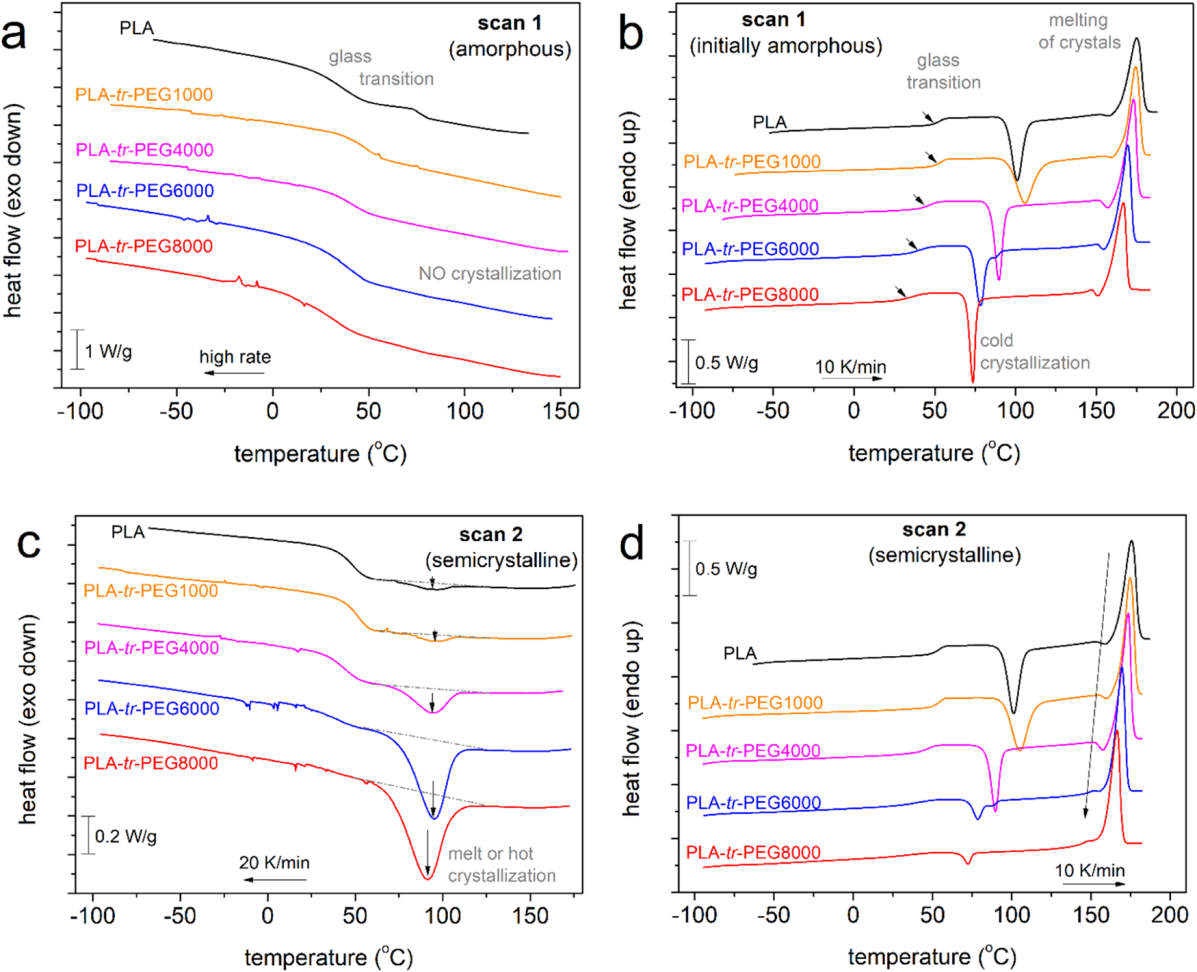
Comparative DSC traces for all samples and scans, being described in the plots, namely, (a, b) cooling and heating during scan 1 and (c, d) cooling and heating during scan 2. In (a), the phrase ‘high rates’ refers to cooling and corresponds to the nonlinear *T* (time) cooling with rates varying from ∼100 to ∼60 K/min within the expected *T* range of crystallization. The arrows indicate the effects recorded during the various thermal events. The heat flow is presented upon normalization to the mass of each sample.

To preserve the samples in the amorphous state and illuminate any direct structure-related effects on the glass transition, we employed fast cooling (scan 1). The results are shown in Figure 3a,b. On the other hand, in scan 2 (Figure 3c,d), which involves milder cooling, melt/hot crystallization is facilitated. Therein, any structural effects on the glass transition are mainly indirect, arising from the presence of the crystals and the semicrystalline morphology (dense or sparse).^69^

**Figure 4:**
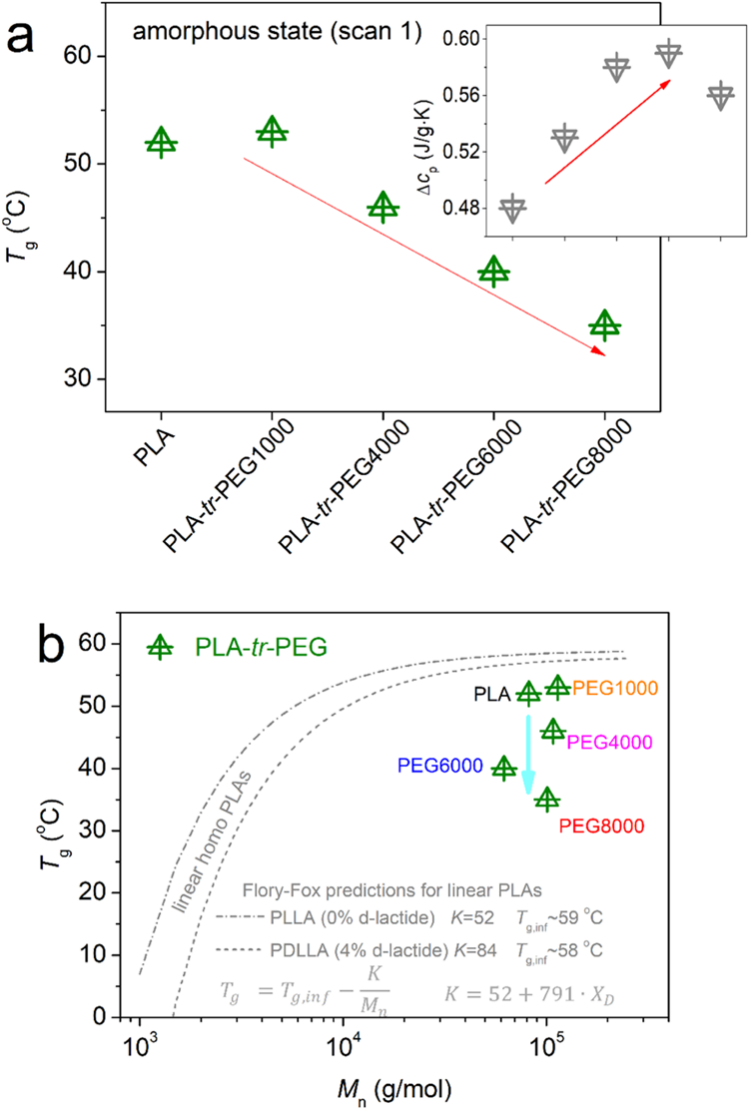
(a) Effects of copolymer composition on the glass transition temperature and Δ*c*_p_ (inset) of all samples in the amorphous state. (b) *M*_n_ dependence of *T*_g_ in the present work compared to the corresponding Flory–Fox predictions in linear PLAs (lines). Adapted with permission from ref (33). Copyright 2024 Elsevier.

The amorphous state of the polymers in scan 1 is confirmed by the elimination of crystallization during cooling (Figure 3a). During the subsequent heating, as shown in Figure 3b, single and clear glass transition steps are recorded for all samples. The characteristic temperature, *T*_g_, was estimated by employing the half heat capacity change (Δ*c*_p_/2) method. The values are included in Table 1 and are also shown in Figure 4a. In neat PLA, *T*_g_ = 52 °C, slightly increases by 1 K in PLA-*tr*-PEG(1000), drops to 46 °C in PLA-*tr*-PEG(4000), and drops further to 40 and 35 °C in PLA-*tr*-PEG(6000) and PLA-*tr*-PEG(8000), respectively. Parallel to this, the strength of the glass transition, Δ*c*_p_, of PLA exhibits a significant increase in the triblocks. These effects suggest that in the triblocks, the segmental dynamics are accelerated and enhanced, which can be interpreted in terms of both chains’ diffusion under fewer constraints and increased freedom of motion.

The mobility of PEG by itself is expected to be much ‘faster’ as the corresponding *T*_g_ is much lower (from about −80 to −60 °C).^70,71^ One would suggest that the presence of the “softer” PEG phase in the “harder” PLA is expected to reduce the overall *T*_g_. However, we should not ignore the extremely small amount of PEG here (PEG/LA = 1/640 ∼ 0.2%). Thus, the results of the increased mobility should be seeked from the copolymer structure, for example, the chain lengths, which are well represented by *M*_n_. In Figure 4b, we plot various *T*_g_ values for the present samples as a function of *M*_n_. At the same time, we compared our results with the *T*_g_(*M*_n_) trends for neat linear PLAs, represented by Flory–Fox predictions confirming experimental findings by DSC in various PLAs being prepared in various ways, including ROP.^27,33^ It is obvious that while PLA and PLA-*tr*-PEG(1000) follow the *T*_g_(*M*_n_) trend of neat PLAs well when increasing the initial PEG length, the corresponding *T*_g_s drop significantly, despite the nonsignificant changes in the *M*_n_. We should note that all *M*_n_ here lay much above the threshold of chain–chain entanglements for PLA (i.e., between 10 and 30 kg/mol).^27,33^ So, to rationalize the suppressed *T*_g_ and elevated Δ*c*_p_, it is most probable that the PEG blocks at the middle of the triblock chains, for *M*_n_ > 1000 kg/mol, impose a plasticization effect.^72,73^

We will attempt to search for further support for the latter by molecular dynamics.

### 3.3 Molecular Dynamics in the Amorphous State

**Figure 5:**
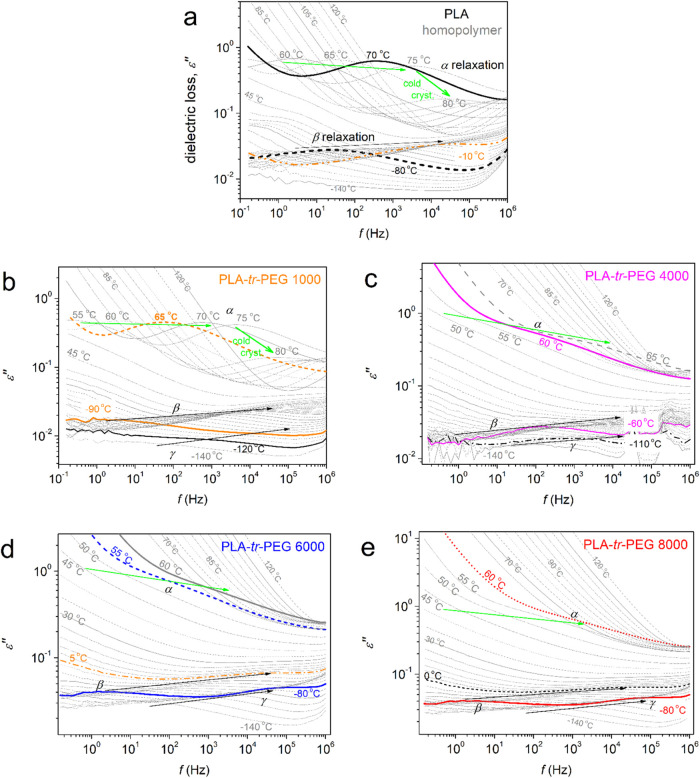
Raw BDS results in the form of isothermal frequency, *f*, and dependence of the imaginary part of dielectric permittivity, *ε″*, for (a) neat PLA and (b–e) PLA-*tr*-PEG copolymers. The basic relaxation processes recorded (peaks) at selected temperatures are marked with arrows.

Molecular mobility is studied here using BDS. This sophisticated technique is widely considered to ‘follow’ the molecular mobility of both segmental (large scale, at *T* ≥ *T*_g_) and local (short scale, *T* < *T*_g_) motions in polymers, via the corresponding dipolar relaxations.^74,75^ This is achieved by recording the maximization of the dielectric loss [*ε*″(*f*,*T*)]. In Figure 5, we present the isothermal plots of *ε*″(*f*) at various *T* values (raw BDS recordings), wherein a variety of *ε*″(*f*) peaks are recorded. The corresponding peak frequencies are related to the relaxation times, τ_rel_, of the dipole moments arising from the actual molecular motions. When *T* increases, the kinetic energy increases, thus, τ_rel_ decreases. Consequently, the relaxation frequencies increase and the *ε*″(*f*) peaks migrate gradually toward higher frequencies. This is the de facto experimental recording of “molecular dynamics”.

The results in Figure 5 were analyzed using a widely adopted fitting method.^74,75^ In particular, the *ε*″(*f,T*) data consisting of mainly complex spectra, wherein more than one relaxation peak coexist, were critically analyzed by fitting model functions, such as the symmetric Cole–Cole (CC) for local dynamics and the asymmetric Havriliak–Negami (HN) for segmental relaxation. At higher temperatures and lower frequencies, where the contribution of ionic conductivity and surface polarization phenomena is significant, additional linear functions and CC were, respectively, employed. For the sake of briefness, we do not present examples from the process of this analysis, as it is a quite trivial process and can be extensively seen in the literature.^33,58,67,76^

**Figure 6:**
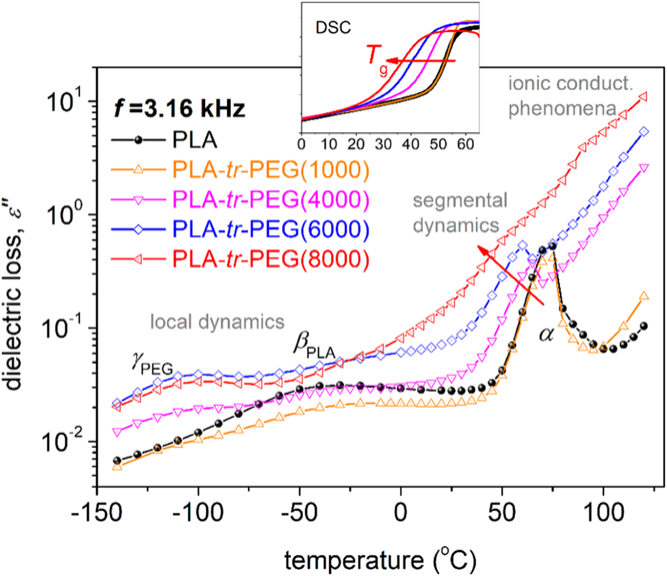
Comparative isochronal BDS curves of *ε″* for all samples at a selected frequency of ∼3 kHz. The identified local (γ_PEG_ and β_PLA_) and segmental (α) relaxation processes are indicated, whereas the added arrow marks the effect imposed on α by the increasing *M*_n_ of PEG in the copolymers. The inset shows the corresponding data for glass transition, as recorded by DSC in scan 1.

To provide an additional way of presentation, and to facilitate a better comparison with the calorimetric recordings in the temperature domain, the BDS results in Figure 5 can be replotted as “isochronal” curves of *ε*″(Τ) at selected frequencies. Such an example of replotting is presented in Figure 6, comparatively for all samples at a relatively high frequency. At low temperatures, below *T*_g_, the dipolar response is due to local motion. In neat PLA, this is the local β_PLA_ relaxation, which is considered to originate from relaxing dipole moments due to rotations (crankshaft motions) of nonbound ester groups (−C=O) of PLA at the polymer backbone.^77,78^ In the PLA-*tr*-PEG systems, an even faster relaxation is recorded (Figures 5, 6). Upon the results by analysis, which follow, and comparison with results from the literature,^79−81^ this is due to the most localized motions of PEG, named γ_PEG_ relaxation^80,81^ or, in other works, being named as the β relaxation of PEG or of poly(ethylene oxide).^79^ Please note that in the past, in PEG^80,81^ and PEG-rich PLA-*tr*-PEG systems,^58^ additional local-like processes were recorded. This is the case of the so-called Johari–Goldstein process^82^ of PEG/PEO, which is also connected to the segmental dynamics. This process could not be reliably resolved in our case, most probably due to the weak contribution of PEG in the expected temperature/frequency region, being dominated by the response of PLA. Regarding segmental dynamics, in the raw data as well as upon analysis, single α relaxation is recorded for all systems. This is an additional fact supporting the homogeneity of the copolymers. The α relaxation, i.e., the dielectric analogue of the glass transition of PLA and PLA-*tr*-PEG(1000), is a strong and narrow peak, in addition to being well separated from the ‘ionic conductivity/interfacial polarization’ phenomena,^67,76,83^ as shown in Figures 5 and 6. In the rest of the copolymers, the relaxation is somehow wider in terms of relaxation times and less discerned from the ionic phenomena.

**Figure 7:**
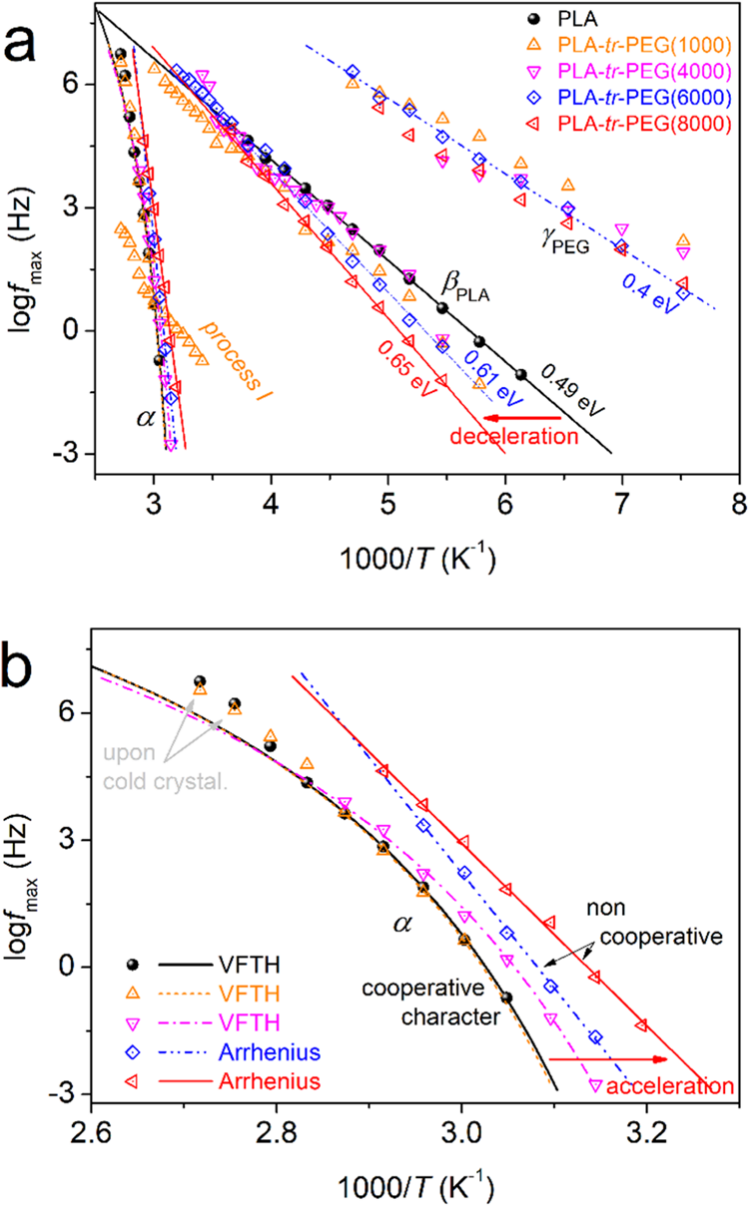
Dielectric relaxation map for all samples (a) in the overall temperature range and (b) focusing on segmental dynamics. The straight and curved lines crossing the experimental points correspond to the Arrhenius and Vogel–Tammann–Fulcher–Hesse equations, respectively.

**Figure 8:**
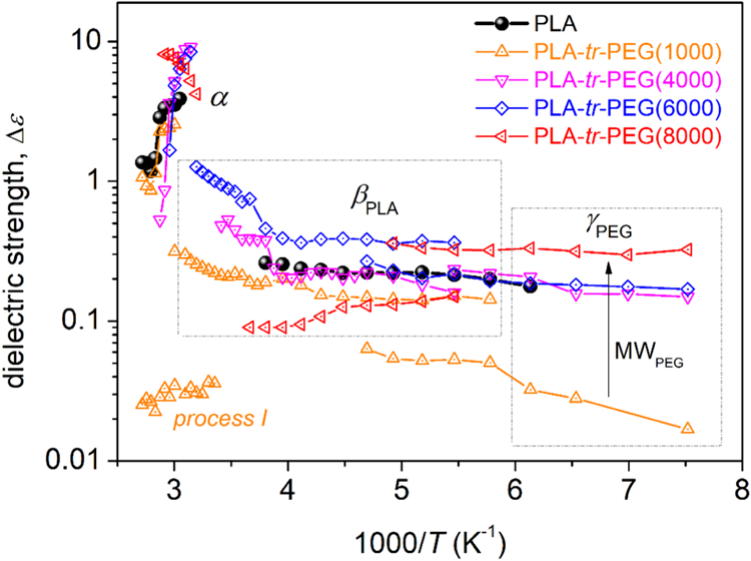
Reciprocal temperature dependence of the dielectric strength, Δ*ε*, for all samples and recorded relaxations.

Despite these, it is clear, e.g., in Figure 6, that the α of PLA migrates toward lower temperatures in the presence of PEG. The result is in accordance with the calorimetric findings for the glass transition (inset of Figure 6). To gain deeper insight, in particular, on the time scale of local and segmental dynamics, we performed a critical analysis of the complex BDS *ε*″(*f*,*T*) spectra adopting widely used routes.^74,75^ In particular, we fitted special mathematical model functions (here, the asymmetric Havriliak–Negami and symmetric Cole–Cole functions) for each relaxation peak. Examples and details of such fitting can be seen in previous works,^33,58,84^ whereas here, we directly present the constructed dielectric relaxation maps. The relaxation map is presented in Figure 7, in terms of the reciprocal temperature, 1000/*T*, and the dependence of the relaxation frequency maxima, *f*_max_, for all resolved processes. The corresponding dependence of the evaluated dielectric strength, Δ*ε*, is presented in Figure 8.

As expected, the local relaxations are located on the right side of the map, i.e., at lower temperatures/higher frequencies. Also, the β and γ relaxations exhibit linear-like time scales. In terms of models, this is the typical Arrhenius behavior^74^ of local dynamics, denoting the temperature-independent activation energy, *E*_act_, of such mobilities. Regarding Δ*ε*, in Figure 8, both β and γ exhibit an increasing Δ*ε* with *T*, which is the expected behavior for local mobility.^74^ Exception to that is the case of β in PLA-*tr*-PEG(8000). Also, in the same figure, we may see that the overall strength of γ interestingly increases with the increase of PEG block lengths. The fastest γ_PEG_ relaxation exhibits nonworth-noting alternations in each time scale between the different copolymers, in addition to an average *E*_act_ of ∼0.4 eV. In PLA-*tr*-PEG(1000) and in the region of segmental relaxation, an additional weak process (I in Figure 7a) was found to be necessary for the best fitting. It is not clear to us whether *process I* is due to actual molecular motion.

On the other hand, the β relaxation of PLA is significantly affected by the presence of PEG in the triblocks. In particular, with the addition of PEG and the simultaneous increase in the length of PEG, β seems to monotonically decelerate while its *E*_act_ increases from 0.49 eV (PLA neat) to 0.65 eV (PLA-tr-PEG8000). Regarding the fitting parameters of each shape (Havriliak–Negami α_HN_ and β_HN_^74^) and, equivalently, on the width of the relaxation times, this is practically similar for all samples (α_HN_ ∼ 0.3, β_HN_ = 1). An exception is PLA-*tr*-PEG8000, which demonstrates a moderately narrow width (α_HN_ ∼ 0.4–0.5, β_HN_ = 1). The wide or narrower relaxation times are usually rationalized in terms of low or higher homogeneity in τ_rel_, respectively.^75^ The overall effects on β in the copolymers could suggest the presence of constraints in the crankshaft rotation of the ester groups of PLA. This can be caused by either their implementation within interactions (e.g., interchain interactions) and/or due to constraints on the PLA backbone segments. Interestingly, both explanations are opposite to the findings of FTIR spectroscopy on the freedom of the ester bonds (Figure 2a) as well as the freedom of motion of the polymer chains (lowering of *T*_g_ in Figure 4, accelerated α relaxation Figures 6, 7). Taking a more careful look at Figure 7a, the acceleration of β does not occur in parallel for all experimental points; moreover, there seems to be a crossover of the various points at higher temperatures (at 1000/*T* ∼ 3.5). Such behavior has not been observed before for PLA or other polyesters. However, seeking similarities in the literature, such an effect was observed in the dynamics of short flexible polymer chains (silicone) and water molecules adsorbed on solid surfaces via relatively strong interactions.^85^ Thus, the alternations here could probably involve interactions; nevertheless, the origins of the peculiar effects on β are in vogue for the time being.

Finally, the focus is on segmental relaxation. In Figure 7b, we replot the time scale points of α relaxation for all samples. Therein, the α relaxation in PLA (bulk) is recorded in accordance with previous cases^33,77,86^ (and references therein), exhibiting a curved time scale. The latter characteristic is indicative of bulky cooperative dynamics and is mathematically expressed by the so-called Vogel–Fulcher–Tammann–Hesse (VFTH) equation^74,87^ (curved lines in Figure 7b). Coming to the triblocks, the α relaxation in PLA-*tr*-PEG(1000) barely changes, whereas it gradually accelerates with increasing initial length of the PEG blocks. Interestingly, the curved time scale (VFTH behavior) is preserved for PEG(1000) and PEG(4000), while it changes to linear-like (Arrhenius behavior) for PEG(6000) and PEG(8000). For the latter two samples, the effects suggest severe suppression of the cooperative character. The effect is quite striking, considering the nonsignificant alternation in the *M*_n_ of the copolymers combined with the drop in *T*_g_ (facilitated chain diffusion).

Regarding the dielectric strength of α, we may follow from Figure 8 that, initially, Δ*ε* decreases with temperature. This is the usual behavior for bulk-unaffected segmental mobility.^33,74,75^ For higher temperatures and with the implementation of cold crystallization, Δ*ε* exhibits a sharper drop, which is followed by a moderate increase at even higher temperatures. With the implementation of cold crystallization, a significant fraction of the polymer chains is immobilized within the crystals, whereas the majority of the amorphous chains suffer constraints by the neighboring crystals, especially within condensed semicrystalline regions. With a further temperature increase, the latter constraints are somehow loosened, and the Δ*ε* (and polarizability) of α increases. Again, PLA-*tr*-PEG(8000) shows exceptional behavior as its Δ*ε*(Τ) dependence monotonically increases. As discussed in the following, the exception behavior in Δ*ε* is connected to the extreme case of the quite ‘fast’ and, simultaneously, “nonfragile” segmental relaxation.

**Figure 9:**
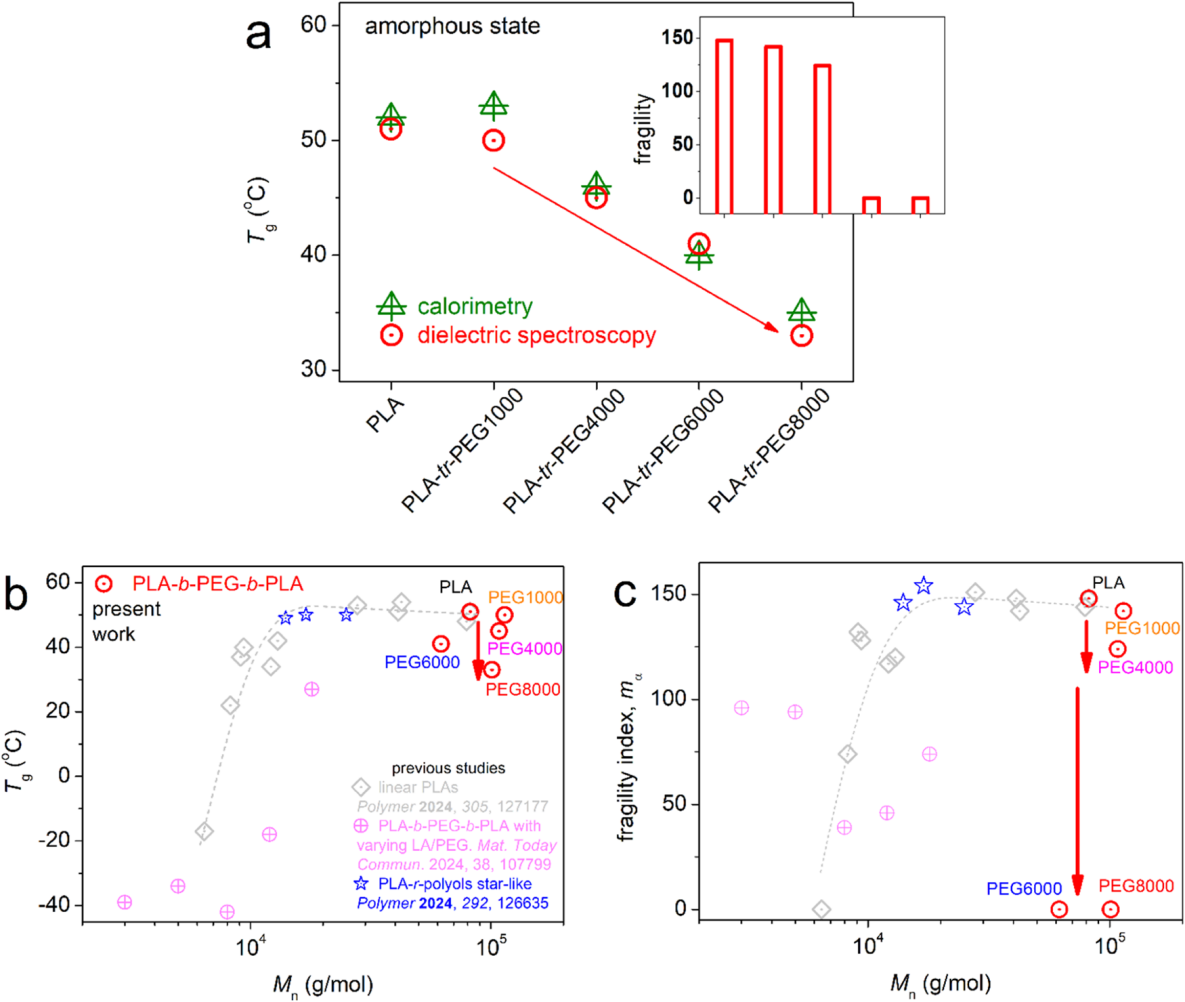
(a) Composition effects on the glass transition temperature, as estimated by DSC and BDS, in the initially amorphous state of the copolymers and neat PLA. The inset shows the corresponding effects on the fragility index of α relaxation, *m*_α_. (b, c) Molecular weight effects on the dielectric *T*_g_ (b) and the fragility index (c) in PLA-*tr*-PEG here, compared with data from the literature on systems prepared using similar ROP processes, namely, linear PLA homopolymers,^33^ PLA-*tr*-PEG with various PLA/PEG ratios,^58^ and star-like PLA-polyols.^84^ Reproduced with permissions from refs (33,58,84). Copyright 2024 Elsevier.

Coming back to the time scale, based on the VFTH fitting and proper parameter fixing, we may estimate two important parameters, i.e., the dielectric glass transition temperature, *T*_g,diel_, and the fragility index, *m*_α_.^33,87^ In simple systems, such as neat polymers, *m*_α_ can be used as a measure of the chains’ cooperativity, being connected to the nanometric cooperativity length, ξ.^88,89^ The extracted *T*_g,diel_ and *m*_α_ values are plotted and are shown in Figure 9a. First, *T*_g,diel_ exhibits a trend quite similar to that of calorimetric *T*_g_. Then, the monotonic drop of *T*_g_ with the increasing PEG block length is accompanied by a parallel drop in *m*_α_ (inset of Figure 9a) from 148 monotonically down to 0. To check the connection of that behavior with the overall *M*_n_, we plotted our data for *T*_g,diel_ and *m*_α_ as a function of the total *M*_n_ of the copolymers, respectively, in Figure 9b and c. For comparison, we included corresponding data for PLA-based materials prepared by similar ROP in these figures. More precisely, we compared with linear PLA homopolymers,^33^ PLA-*b*-PEG-*b*-PLA triblocks with varying LA/PEG ratios,^58^ and star-like PLAs.^84^

The comparison employed is quite illuminating. PLA and PLA-*tr*-PEG(1000) demonstrate bulk-like dynamic dependences from *M*_n_, similar to those of neat PLAs. On the other hand, for PEGs(4000–8000), both *T*_g,diel_ and *m*_α_ were below the linear PLA trends, with minor effects of *M*_n_. *M*_n_ seems to play a more significant role within different systems, for example in linear, star-like, and triblock with significantly shorter lengths, namely, for *M*_n_ mainly below the expected entanglement threshold.^33^

To rationalize the situation herein, we may employ a simple scenario and a more complex one. In the simple scenario, the overall results suggest that the easier diffusion of chains and suppressed cooperativity could be due to the aforementioned plasticization induced by PEG. In this context, plasticization leads to increased free volume^90^ and, therefore, to, on average, detachment of neighboring triblock entities throughout the copolymer volume. The detachment is compatible with either the cooperation of chains within a wider ξ or/and the cooperation of fewer chains in a given volume. Such a scenario was successfully employed in previous works on neat polymers^33,69^ and similar PLA-*tr*-PEGs.^58^ In the latter triblocks, the phenomena were actually stronger, as quite more PEG was involved in addition to stronger suppression of the overall *M*_n_.

The complex scenario that could be speculated here could involve partial formation of more complex forms in the copolymers. For example, the triblocks could be formed/organized in micelle-like entities, with some PEGs located at the “central shell” of the micelle and the PLA blocks located around it forming the “outer shell”.^59,60^ Despite this being in accordance with the proposed “increased free volume from the point of view of PLA”, it lacks firm manifestation. Hopefully, we will be able to address this point in our future study, keeping in mind that these systems are promising for demonstrating such self-organization in certain environments (aqueous, pH, temperature).^59^

### 3.4 Crystallization Aspects

**Figure 10:**
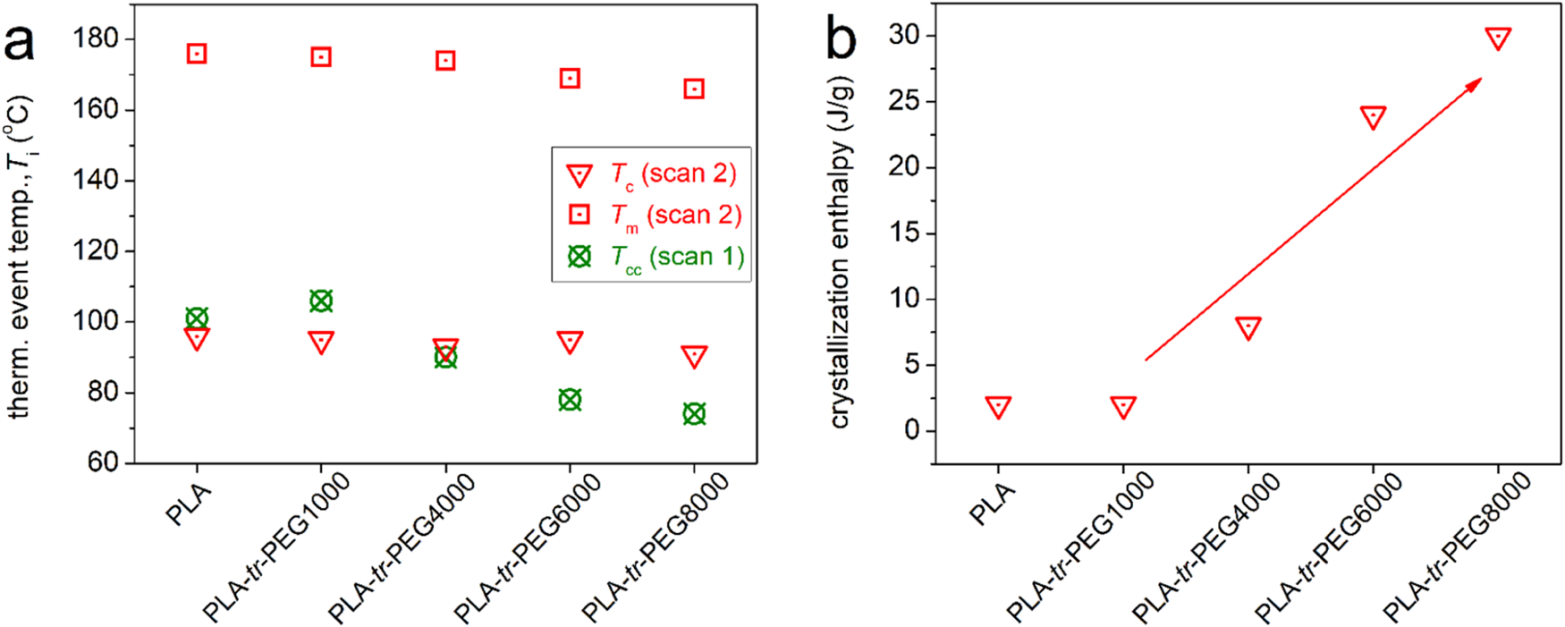
Effects on (a) the characteristic temperatures of crystallization and melting and (b) the enthalpy change of melt crystallization.

In this section, we will discuss aspects related to crystallinity based on the results of calorimetry, X-ray diffraction, and polarized microscopy. Regarding calorimetry, in Figure 3c (scan 2), all samples exhibited melt crystallization at temperatures, *T*_c_, between 91 and 96 °C, with increasing enthalpies, Δ*H*_c_, varying from 2 to 30 J/g. Comparing the measured Δ*H*_c_ with the heat of fusion for PLA, Δ*H*_100%,cryst,PLA_, taken as 93 J/g,^91^ the corresponding crystalline fraction (*X*_c_ = Δ*H*_c_/Δ*H*_100%,cryst,PLA_) varies from ∼2 to ∼32 wt %. In Figure 10a, we may observe that regarding melt crystallization (scan 2), *T*_c_ exhibits mild alternations, with a weak tendency to decrease from PLA to the copolymers. Similar weak effects are recorded for the melting temperature *T*_m_ (166–176 °C). We may then conclude that the necessary supercooling (*T*_m_–*T*_c_) for the evolution of crystallization from the melt state is similar for PLA and PLA-*tr*-PEG. The effect can be understood in terms of unaffected nucleation.^92^ Despite that, as shown in Figure 10b, the Δ*H*_c_ of PLA exhibits a strong increase in PLA-*tr*-PEG(4000–8000), with the latter being monotonic when increasing the *M*_n_ of PEGs from 4000 to 8000. Combined with the constant nucleability and enhanced mobility of the mobile amorphous chains (lowering of *T*_g_), we may conclude that in the copolymers, the spherulites grow faster, if not larger, as compared to PLA and PLA-*tr*-PEG(1000).

A similar conclusion can also be made regarding cold crystallization. The “pure” cold crystallization can be better followed during the heating of scan 1 (Figure 3b). Therein, cold crystallization is recorded at *T*_cc_ between 74 and 101 °C, with *T*_cc_ of PLA and PLA-*tr*-PEG(1000) being quite similar but being suppressed “systematically” when increasing the length of the PEG block. Considering the similar nucleation between the samples, the drop in *T*_cc_ is most probably related to the enhanced chain diffusion,^92,93^ leading to faster (i.e., at lower *T*) crystal growth.

**Figure 11:**
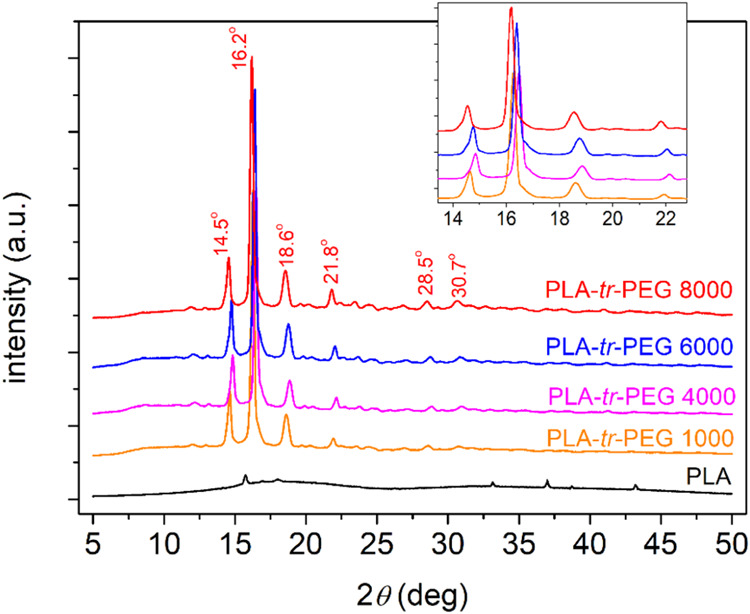
Comparative XRD patterns of all samples subjected to melting and subsequent cooling at 20 K/min.

To make the study of crystallization more complete, we employed XRD on samples subjected to melt crystallization thermal treatment, similar to that used in DSC scan 2. The comparative XRD spectra are shown in Figure 11. Therein, neat PLA barely exhibits crystalline diffraction peaks; thus, the result suggests that it is mainly a highly amorphous polymer. A significant number of strong diffraction peaks are recorded within all triblocks. This is quite impressive and is, in general, in accordance with the calorimetric findings of Δ*H*_c_ in Figure 10b. The 2θ positions of the crystalline peaks, marked for a selected sample, do not show systematic alternations, neither in the position nor in the number. Comparing with previous findings on PLA,^41,94,95^ it seems that the crystals formed here are mainly of the *α*′ type of PLA.

**Figure 12:**
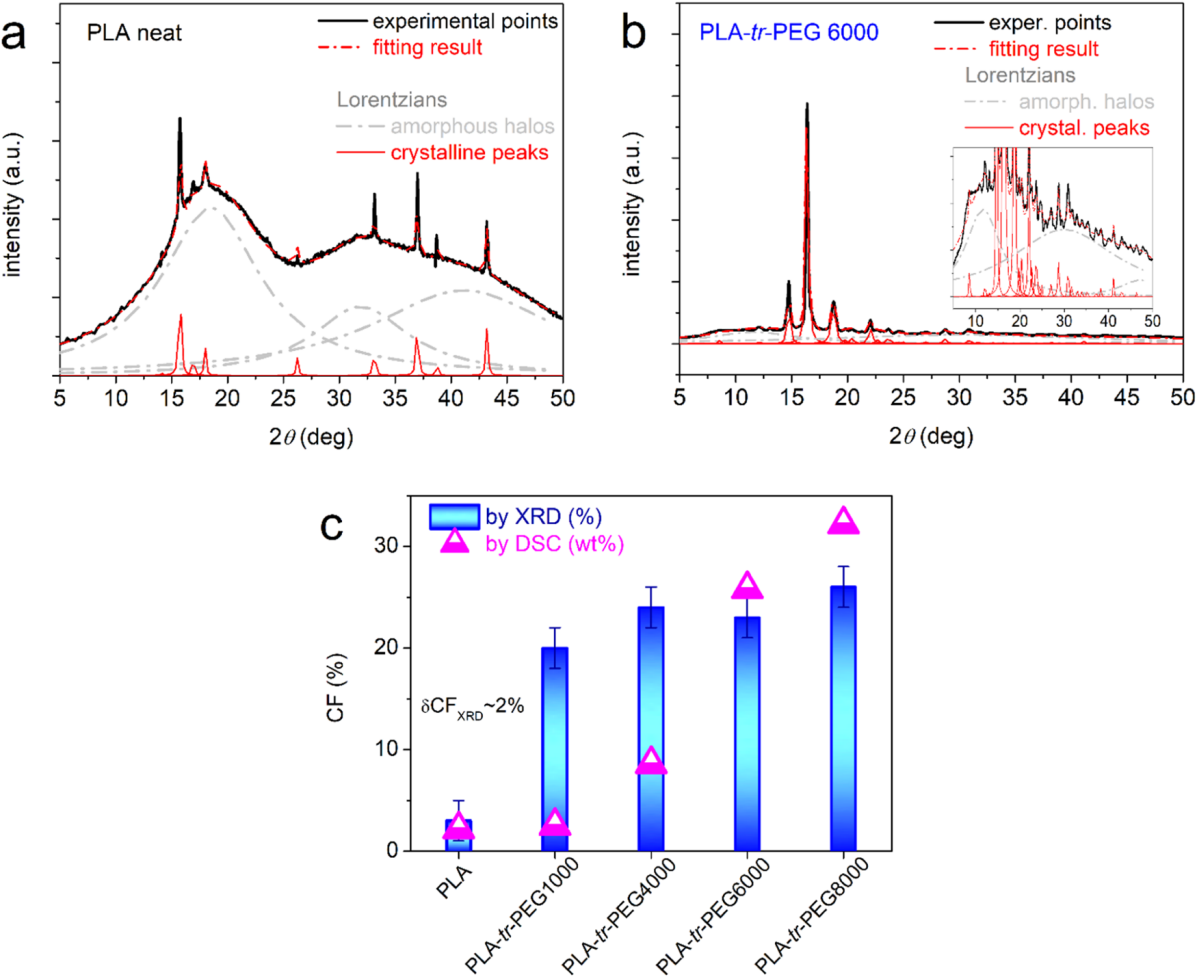
(a) and (b) Examples of the analysis of complex XRD spectra in terms of mathematical model functions. (c) Diagrams of the crystalline fraction estimated by XRD (columns) and DSC (up-pointing triangles) for all samples.

The XRD spectra were analyzed by fitting^96^ Lorentzians model functions for both the amorphous halos and the crystalline peaks. Two examples of these fittings are shown in Figure 12a and b. Adopting a known route, we could estimate the degree of crystallinity via XRD and CF_XRD_ by the comparison of the sum of the crystalline diffraction areas with the total diffraction (amorphous and crystalline).^97^ The effects on CF_XRD_, along with the uncertainty estimated by the fitting method (±2%), are plotted as column diagrams in Figure 12c. The quite low crystallinity of PLA (∼3%) is strongly enhanced in the triblocks (20–26%). Qualitatively, the trend is similar to the calorimetric findings (CF by DSC in Figure 12c), with the main difference located in the case of PLA-*tr*-PEG(1000) and, in general, in the absolute values. Most probably, the quantitative difference is due to the different in-principle techniques, as well as the fact that the CF_DSC_ is estimated by assuming that the PLA chains uniquely contribute to the crystal formation. In the case of the copolymers this is, for sure, not the case.

**Figure 13:**
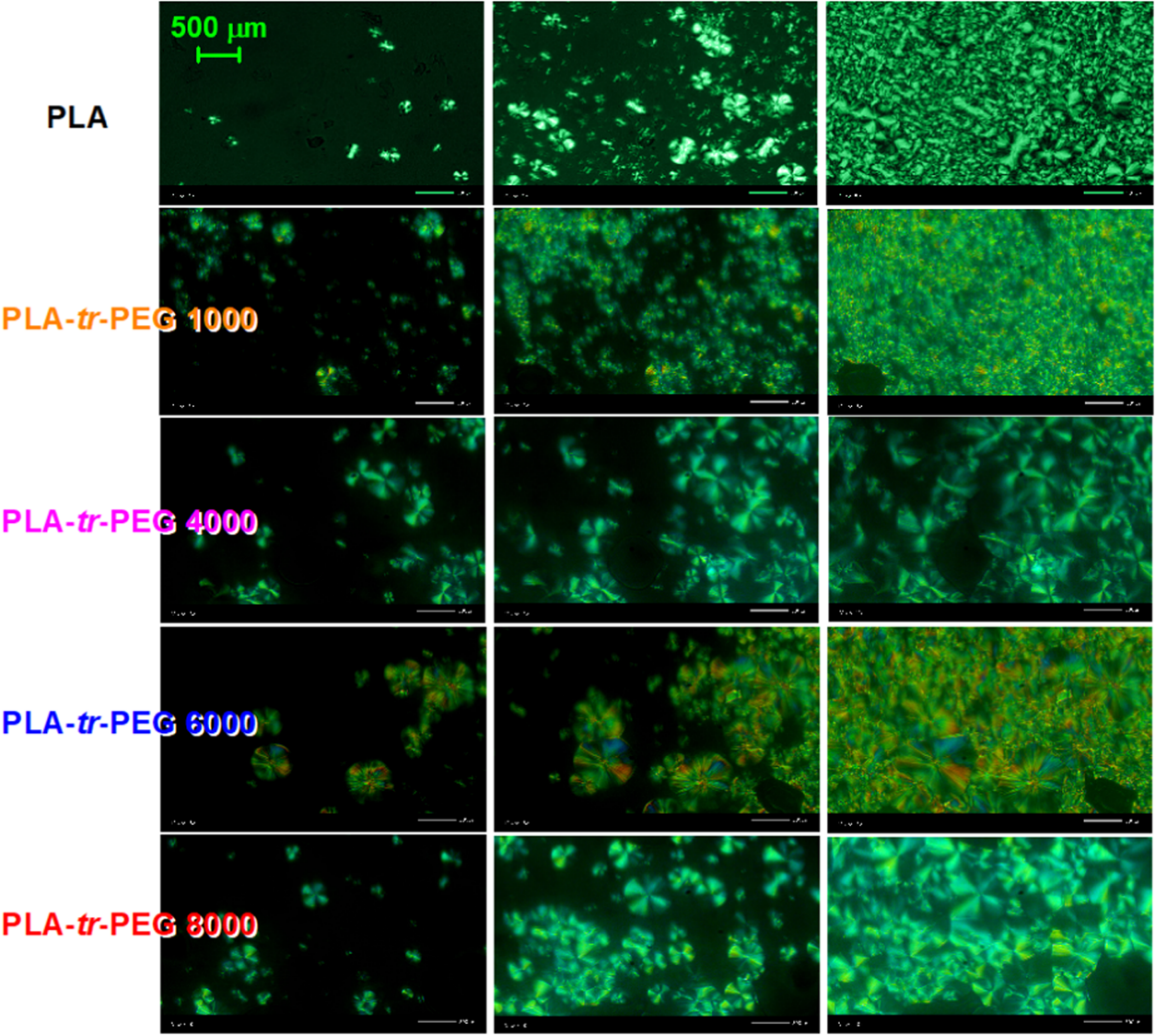
PLM micrographs of all samples during melt crystallization. The images shown are snapshots of the initial (left), intermediate (middle), and final (right) stages of the crystallization.

To complete the view of crystallization, PLM measurements were performed, in particular, on the evolution of crystallization beginning from the melt state and during subsequent cooling. The selected PLM micrographs during the initial, intermediate, and final crystallization views are presented in Figure 13.

Beginning with the initial stages (left side of Figure 13), it seems that a similar number of crystallites were formed for all samples. This roughly confirms the calorimetric recording of nonaltered nucleation. Despite this, it is clear that the size of the crystals, at the initial stage as well as the final view (right side of Figure 13), is smaller in PLA and PLA-*tr*-PEG(1000) and larger (almost doubled) in the PLA-*tr*-PEG(4000–8000) copolymers. The result again supports the conclusions extracted by DSC on the significant role of enhanced chain diffusion in the case of the triblocks, driven by the overall plasticization role of PEG on PLA. Obviously, we expected and confirmed by the PLM data that any antagonistic effects between the fast/slower growing of crystals around neighboring nuclei will be suppressed in the triblocks due to the lower *T*_g_ in the copolymers and the increasing distances “*T*_c_–*T*_g_” and “*T*_m_–*T*_g_”.^98^ As a result, in the final semicrystalline state, we record in PLA-*tr*-PEG(4000–8000) spherulites larger in size and smaller in number than those in neat PLA and PLA-*tr*-PEG(1000), which exhibit similar views.

These systematic alternations in the semicrystalline morphology, chain diffusion in the amorphous part, and in free volume (plasticization) are parameters known to relate to the material properties, i.e., from the mechanical^44^ to permeability^99^ aspects. Therefore, considering the envisaged biomedical applications and renewability of our triblocks, as a next step and our ongoing work, we try to correlate the present findings with the macroscopic performance of PLA-*tr*-PEG (enzymatic degradation, mechanical properties, solubility, thermoresponsive transitions in aqueous environments, etc.).

## 4 Conclusions

The newly prepared series of triblock PLA-*b*-PEG-*b*-PLA copolymers, with a fixed low PEG/LA molar ratio of 1/640 and altered length of the initial PEG block (from ∼1000 to ∼8000 g/mol), was studied from the point of view of thermal transitions and local and segmental molecular dynamics. The employed synthetic route, involving *in situ* ROP over existing PEG blocks, resulted in homogeneous systems with no significant variations in the overall molecular weight, contrary to previously studied similar systems of fixed PEG blocks and altered PEG/LA ratios. Comparing the latter systems^58^ and various ROP-synthesized neat linear PLA homopolymers,^33^ herein, the strong plasticization effect of PEG on PLA was clearly evidenced. The conclusion was based on the glass transition dynamics enhancement and suppression of the cooperativity degree of chains, with the length of the PEG block being found to be the critical parameter here. In particular, for PEG1000 (the shorter one), the behavior of the triblock was found to be similar to that of neat PLA, whereas, for PEG-4000 up to PEG8000, *T*_g_ was found to systematically drop by more than 15 K. This is quite striking considering the small amount of PEG (∼2‰). The combination of faster segmental dynamics and estimated increased free volume in the triblocks, always compared to the homo-PLA, imposed a significant impact on crystallization. More precisely, the weak crystallinity degree of PLA (2–3%) increased up to 20–26% in PLA-*tr*-PEG, whereas nucleation was found to be mainly unaffected. This combination resulted in altered semicrystalline morphology in the triblocks, namely, the formation of larger spherulites (almost double in size) but fewer in number, which in all cases seem to fill the polymer volume. It is expected that the alternations in *T*_g_, free volume, semicrystalline morphology, etc., will introduce severe changes in the final material’s macroscopic properties (mechanical, permeation, enzymatic hydrolysis, and biodegradation) to a wide extent. Considering the future applications for these systems, such as, in cosmetology or as drug carriers, it is desirable to choose the proper material (performance) for the desired use, a potential being provided by these and similar PLA-based systems. Besides the applications, in this work, we demonstrate once again that the combination of results from carefully conducted measurements and proper analysis can provide “indirect” quite in-depth information on the structure–property relationships in polymers, which cannot be easily assessed by direct observation (e.g., microscopes).

## Data Availability

The raw/processed data required to reproduce these findings cannot be shared at this time due to technical or time limitations.
